# The Neural Mechanisms of Meditative Practices: Novel Approaches for Healthy Aging

**DOI:** 10.1007/s40473-016-0098-x

**Published:** 2016-10-18

**Authors:** Bianca P. Acevedo, Sarah Pospos, Helen Lavretsky

**Affiliations:** 1 Semel Institute for Neuroscience and Human Behavior, UCLA, Los Angeles, CA USA; 2 Neuroscience Research Institute, University of California, Santa Barbara, Santa Barbara, CA 93106 USA

**Keywords:** Meditation, Yoga, Tai Chi, Qi Gong, Mindfulness, Brain, Aging

## Abstract

**Objectives:**

Meditation has been shown to have physical, cognitive, and psychological health benefits that can be used to promote healthy aging. However, the common and specific mechanisms of response remain elusive due to the diverse nature of mind–body practices.

**Methods:**

In this review, we aim to compare the neural circuits implicated in focused-attention meditative practices that focus on present-moment awareness to those involved in active-type meditative practices (e.g., yoga) that combine movement, including chanting, with breath practices and meditation.

**Recent Findings:**

Recent meta-analyses and individual studies demonstrated common brain effects for attention-based meditative practices and active-based meditations in areas involved in reward processing and learning, attention and memory, awareness and sensory integration, and self-referential processing and emotional control, while deactivation was seen in the amygdala, an area implicated in emotion processing. Unique effects for *mindfulness practices* were found in brain regions involved in body awareness, attention, and the integration of emotion and sensory processing. Effects specific to *active-based meditations* appeared in brain areas involved in self-control, social cognition, language, speech, tactile stimulation, sensorimotor integration, and motor function.

**Summary:**

This review suggests that mind–body practices can target different brain systems that are involved in the regulation of attention, emotional control, mood, and executive cognition that can be used to treat or prevent mood and cognitive disorders of aging, such as depression and caregiver stress, or serve as “brain fitness” exercise. Benefits may include improving brain functional connectivity in brain systems that generally degenerate with Alzheimer’s disease, Parkinson’s disease, and other aging-related diseases.

## Introduction

For thousands of years, ancient Eastern contemplative practices have been used by humans to expand consciousness, obtain enlightenment, and connect to one’s spiritual nature, as well as to maintain longevity. In recent times in the West, these practices have increasingly been used by millions of people for stress reduction, enhancing well-being, and improving coping with chronic diseases of aging, such as cancer, and various neurodegenerative and cognitive disorders such as Alzheimer’s and Parkinson’s diseases [1•, 2–5]. Growing scientific evidence supports clinical use and efficacy of some interventions with increased understanding of the underlying neurobiological mechanisms [6]. More recently, the cognitive and neuroplastic effects associated with different meditative practices have received significant attention from researchers interested in novel and less invasive ways to enhance cognition, treat stress-related disorders, and prevent cognitive decline.

### The Role of Mind–Body Medicine in the Aging Society

The world’s population over the age of 60 will increase almost twofold, from 901 million to more than 1.4 billion by 2030, and rates are estimated to reach 1 in 6 people [7]. The World Health Organization (WHO) reported that the most years of healthy life are lost due diseases involving hearing and vision impairment, osteoarthritis, ischemic heart disease, dementia, chronic obstructive pulmonary disease, cerebrovascular disease, and depression [8]. Typically, two or more aging and stress-related health conditions co-occur [9], for which the use of polypharmacy is common and increases the risk of drug interactions and adverse reactions to as high as 35–60 and 13–82 %, respectively [8, 10]. The use of inexpensive mind–body interventions, such as yoga or meditation, can help achieve this goal via non-pharmacological stress reduction, brain enhancement exercise, and lifestyle changes.

The US National Health Interview Survey (NHIS) reported the linear growth of mind–body interventions such as yoga, Tai Chi, and Qi Gong among people ages 18 and over. The data from 2002, 2007, and 2012 found that yoga was the most popular approach, accounting for 80 % of these practices. The same surveys showed similarly growing popularity of yoga practice in people ages 65 and over, with the prevalence constantly increasing from 1.3 to 2.2 and 3.3 %, respectively [11]. Therefore, using mind–body approaches may satisfy unmet medical needs by relieving symptoms associated with chronic diseases and pain and by possibly reducing side effects associated with conventional drugs [12]. The use of complementary and integrative medicine (CIM) could supplement conventional medicine in the management of mild–moderate mental and physical symptoms (e.g., pain, depression, anxiety, insomnia, etc.) [13–17]. It may be beneficial for treatment and prevention of aging-related disorders because of the relatively low-cost and improved safety profile.

### Mind–Body Clinical Research

Research aiming to understand the mechanisms underlying mind–body therapies has grown substantially in recent decades. Although many sophisticated techniques and studies are taking place to examine the neural and other physiological targets affected by mind–body practices, this body of research still faces some major challenges. One issue stems from the myriad of mind–body practices described throughout history in the East, and now practiced by Western practitioners, including meditation, yoga, Tai Chi, and Qi Gong. Some of the yoga practice “brands” have become more popular in the West with millions practicing “hot” (or Bikram), Vinyasa, Hatha, Iyengar, and Kundalini.

Similarly, there are many types of widely used meditations that include varied attention-focused practices such as mindfulness-based stress reduction (MBSR), Vipassana, and Zen meditation [18••], and less known types including loving-kindness Meditation (LKM) [19•, 20] and integrative body–mind training (IBMT). Other type of mind–body practices include “moving meditation” or mindful physical exercise, like yoga, which incorporates anatomical alignment and postures (asana) with certain hand positions (mudras) and breath work (pranayama).

Researchers have attempted to classify meditation techniques according to the focus of attention. For example, some have distinguished between focused-attention (FA) meditation, a method of concentrating attention on a particular stimulus, versus open monitoring (OM) where practitioners are instructed to focus on all incoming stimuli, internal sensations, and thoughts while maintaining a state of present and non-judgmental awareness [21]. Somewhat similarly, Newberg and Iversen (2003) [22] distinguished between guided meditation, where the practitioner is led to focus on a particular object, image, or phrase or guided in visualization, versus volitional meditations, where individuals practice the meditation by their own will. Researchers have also distinguished between internally focused meditations, where the goal is for the practitioner to clear themselves of thoughts to reach a meditative state, versus externally focused meditations, where the practitioner’s attention is placed on something outside of the self, such as a mantra.

Expanding on these models, Wang and colleagues (2011) [23] delineated between “focused-based” and “breath-based” meditation practices. In one study, they examined cerebral blood flow (CBF) differences among experienced Kundalini Yoga (KY) practitioners (over 30 years of experience) while doing a focused-based meditation versus a breath-based meditation. The results showed both unique and common activations for the two meditation types, such that a focused-based meditation showed greater activation of neural areas involved in attention, such as the prefrontal cortex and the cingulate gyrus.

Our own recent study examined the effects of KY and Kirtan Kriya meditation compared to memory enhancement training on functional brain activity among older people (ages 55+ years) experiencing mild cognitive impairment. The results showed that enhanced verbal and visual–spatial memory performance was associated with increased connectivity in the default mode network (DMN) of the brain known to be involved in neurodegenerative disorders and AD [1•].

Clearly, these varying styles of yogic meditation pose a challenge for understanding the detailed mechanisms underlying its effectiveness. There are also individual differences whereby some people report immediate changes in their levels of stress and cognitive performance, while others do not. Self-selection bias poses yet another challenge as studies with experienced meditators may be investigating populations that have an affinity for meditative practices, and thus, results may partly be due to placebo effects associated with “belief” that the practice is beneficial, one’s own initiative, and “liking” of the practice.

Beyond practical and individual differences, the field also faces the challenge of consolidating findings given the wide range of methodologies and research designs that have been used to examine yoga’s effects on the human brain. For example, researchers have applied cross-sectional, longitudinal, and even randomized control studies. They have also compared yoga’s effects across expert meditators and novices, and some studies have examined meditation’s effects on healthy, normative populations, while others have investigated yoga’s effects on specific ailments among clinical and aging samples [24–30]. Research has also varied in measurement types from structural brain differences, to neural activity, and changes in blood flow, as well as the type of task used during brain assessment, from resting-state brain to effects shown during actual meditation. Finally, various control conditions have been implemented in yoga research such as listening to relaxing music, engaging in exercise regimens, focused memory-training classes, and even repeating animal lists.

At this time, the field of contemplative neuroscience is coming to a consensus about potential applications of mind–body practices, consolidating findings given the wide range in methodologies and research design. To date, the data include structural brain differences, change in neural activity using functional MRI (fMRI), and the type of task used during brain assessment, from resting-state brain to meditation-specific changes (fMRI), and changes in blood flow (fMRI) or positron emission tomography (PET) or single-photon emission computed tomography (SPECT).

### Review of the Neural Mechanisms of Yoga and Meditation

In recent years, a number of systematic reviews have identified some overarching conceptual frameworks and neural networks that are useful for understanding how yoga works. For example, a meta-analysis of 21 studies (and roughly 300 meditators) examining the structural brain changes associated with meditation found that approximately eight brain regions consistently showed significant morphological differences [31••]. The neural regions identified were mainly in areas involved in meta (frontopolar cortex/BA 10) and sensory awareness (sensory cortex and insula), attention (anterior and mid-cingulate), memory (hippocampus), and emotion regulation (orbitofrontal cortex). In sum, we can conclude that mainly structural differences resulting from meditation were found in areas that affect awareness, attention, and emotion regulation. Many of these regions are also affected in cognitive and mood disorders, aging-related diseases, and other disorders such as Alzheimer’s and Parkinson’s diseases [32, 33]. In fact, several clinical studies have started to investigate meditations’ effectiveness in enhancing memory and cognitive function in individuals experiencing mild cognitive impairments (MCIs) to more severe forms, such as Alzheimer’s disease [1•, 3, 4, 24]. In sum, structural differences resulting from meditation were found in areas that affect awareness, attention, memory, and emotion regulation; thus, researchers have started to investigate meditation’s effectiveness for alleviating symptoms associated with cognitive, mood, and age-related disorders.

In another meta-analysis, Tang and colleagues (2015) [18••] aggregated mindfulness meditation studies that examined changes in functional activity, neural connectivity, and structural brain changes. They focused their review on meta-analyses and research applying rigorous designs, such as longitudinal studies and randomized control trials with active control groups. They found that six brain regions were consistently affected by mindfulness meditation, mainly areas involved in cognitive processing and executive control (prefrontal cortex); attention (anterior and posterior cingulate); sensory awareness (insula); reward, learning, and motivation (striatum); and emotional processing (amygdala). Based on the known function of these regions, the authors concluded that mindfulness meditation may enhance well-being via the neural circuits that regulate emotion and self-awareness and present-moment awareness. These conclusions echoed those of an earlier review by Holzel et al. (2011) [34], which proposed that mindfulness meditation works by enhancing attention, emotion regulation, body awareness, and self-awareness of a non-judgmental nature.

Although there are some common themes and similar conclusions emerging from extensive reviews of meditation studies, there still seems to be some lack of consensus due to the varying findings of neural networks identified as yoga’s main targets of action. This may also be due to the various exclusion and inclusion criteria used in reviews and meta-analyses. For example, the review by Tang et al. (2015) included only mindfulness-based studies that measured activation or structural changes in the human brain [18••]. However, the review by Fox et al. (2015) [31••] included all meditation types but only of studies investigating structural brain changes. As such, there are a variety of ways to slice up the yoga/meditation research pie, which poses a challenge for reaching a definitive consensus. However, there is sufficient evidence to provide an overarching model of how meditation works as well as a number of nuanced approaches to explore some of the specific effects resulting from different meditative practices.

Other types of findings include examination of neurotransmitters and brain chemistry as targets for meditative practices. Streeter and colleagues (2012) [35] summarized some of the biological mechanisms by which yoga may improve mental and physical health. They hypothesized that environmental stress contributes to imbalances of the autonomic nervous system (ANS), with decreased parasympathetic nervous system (PNS) and increased sympathetic nervous system (SNS) activity. This is associated with underactivity of the GABA system in the brain. Streeter hypothesized that yoga-based practices, and particularly practices that alter breathing patterns, increase activity of the PNS and GABA system in part via the afferent vagus nerves, which are the primary peripheral pathway of the PNS. Indeed, research suggests that yoga increases PNS activity [35] and GABA levels in the thalamus and that these increases are correlated with improved mood [36].

Other research suggests that the benefits of mind–body interventions are relayed via the downregulation of stress, mainly through the hypothalamic–pituitary–adrenal (HPA) axis, known for its role in stress control, and the SNS [24]. Studies have shown that yoga is associated with reductions in plasma cortisol, mainly in clinical samples with depression, those undergoing treatment for breast cancer, or those suffering from addiction to alcohol [25, 26]. More broadly, a review of 81 studies found that yoga surpassed exercise regimens in numerous outcome measures of health such as salivary cortisol, blood glucose, fatigue, pain, and sleep in both healthy and clinical samples. However, there are some studies that did not find associations between yoga and changes in cortisol levels [27–29].

Finally, there is some evidence suggesting that yoga is associated with endogenous dopamine release in the ventral striatum, a major area of the brain’s reward system [37]. Dopamine is a neurotransmitter that is implicated in feelings of pleasure and craving, reward learning, motivation, mood, and responding to novel stimuli [38, 39]. Certainly, physical activity that is rewarding, active, and novel may promote feelings of pleasure and euphoria. Thus, it is important to distinguish between quiet, inwardly focused meditation types and those that involve more active engagement. Moreover, distinguishing between these two forms of meditation may be particularly important for aging populations especially in those with movement disorders, such as Parkinson’s disease.

### The Present Systematic Review

In this focused review, we compare the neural mechanisms of meditative practices that use a single focus of attention, for example, while sitting still and focusing one’s attention internally to quiet the mind (e.g., mindfulness, Zen) versus those that use multiple foci of attention, chanting, active postures (asanas; mudras), breath (pranayama), or working with a partner. Our first goal was to identify commonneural networks affected by both active-based and mindful meditations and to begin to delineate an overarching framework for meditation’s neural mechanisms. Our second aim was to determine the unique neural regions affected by these two styles of meditation to describe the potential specific benefits provided by each approach. Thus, in this review, we first provide a brief outline of the neural networks found to be commonly activated across various meditation styles. Second, we discuss the unique neural contributions of these different meditation techniques and account for possible factors pertaining to these variations. Lastly, we discuss the implications for aging and geriatric populations.

## Method

### Study Selection

The selection process took place in two stages. First, we retrieved all articles reporting on the neural activation and structural effects associated with meditation. We defined meditation as mental training to obtain a relaxed state of present-moment awareness. In this review, we included all studies that focus on such meditative techniques and subsequently categorized them into different meditation styles. Although we focused on systematic reviews, we also included individual studies associated with mindful physical exercise (mostly yoga practices), as there is nearly no available review of this work. Second, we assessed the articles for eligibility and focused on reviews summarizing the effects of meditation on the brain. Next, studies were classified according to sample criteria such as meditation type, expert meditators versus novices, study design, and type of control group.

## Results

A total of 13 studies were identified as fitting our inclusion criteria, seven were systematic reviews of mindfulness-based meditations, and six were studies of active-type meditations. Study and sample characteristics are summarized in Tables 1, 2, and 3.

**Table 1:** Mindfulness-based meditative practice meta-analysis and systematic review studies

Study	Meditation type	Meditation group (M)	Control group (C)	Sample size (*n*) (*M* = mean age)	Brain measurements
Meta-analysis					
Fox et al. (2014) [31••]	Insight Zen BSM Tibetan Buddhist MBSR IBMT BWV Soham LKM	*N* = 21 studies		Approximate M: *n* = 387 C: *n* = 358	1. Cortical thickness and gyrification 2. Gray matter volume and concentration 3. DTI
Systematic reviews					
Tang et al. (2015) [18••]	Insight Zen Tibetan Dzogchen MBSR IBMT MBI	*N* = 12 studies		Approximate M: *n* = 204 C: *n* = 194	1. Cortical thickness 2. Gray matter volume and density 3. Hippocampal volume 4. DTI
Chiesa and Serretti (2010) [40]	Vipassana Zen MBSR MBCT	*N* = 52 studies			1. EEG 2. Brain fMRI
Ott et al. (2011) [41]	Insight Zen Vipassana Tibetan Buddhism Zazen Vipassana Samantha	*N* = 5 studies, expert meditators for 3–24.2 years		Approximate M: *n* = 85 C: *n* = 80	1. Cortical thickness 2. Gray matter volume and concentration
Marciniak et al. (2014) [42]	Zen Vipassana Tibetan Buddhism MBSR	*N* = 9 studies		Approximate M: *n* = 186	1. Cortical thickness

Meditation types: *BSM* body-scanning meditation, *MBSR* mindfulness-based stress reduction, *IBMT* integrative body–mind training, *BWV* brain wave vibration, *LKM* loving-kindness meditation, *MBI* mindfulness-based intervention, *MBCT* mindfulness-based cognitive therapy; measurements: *DTI* diffusion tensor imaging, *EEG* electroencephalogram, *fMRI* functional magnetic resonance imaging

**Table 2:** Mindfulness-based meditation cross-sectional studies

Study	Meditation type	Meditation group (M)	Control group (C)	Sample size (*n*) (*M* = mean age)	Brain measurements	Task at measurement
Cross-sectional studies						
Luders et al. (2015) [43]	Samatha Vipassana Zen Kriya Tibetan Buddhism Raja Yoga Mindfulness Buddhist Dzogchen Sadhana Mahamudra Chenrezig Kundalini	Expert meditators for 4–46 years; healthy		M: *n* = 50 (*M* = 51.4 years) C: *n* = 50 (*M* = 50.4 years)	1. Global and local gray matter (brain MRI.	Control: ICBM database
Newberg et al. (2010a) [44]	MBSR	Expert meditators for ≥15 years; healthy		M: *n* = 14 (*M* = 45 years) C: *n* = 14 (*M* = 43 years)	1. CBF 2. SPECT	t1: rest, eyes closed

Meditation types: *MBSR* mindfulness-based stress reduction; measurements: *MRI* magnetic resonance imaging, *CBF* cerebral blood flow, *SPECT* single-photon emission computed tomography; database: *ICBM* International Consortium for Brain Mapping

**Table 3:** Yoga studies

Study	Meditation type	Meditation group (M)	Control group (C)	Sample size (*n*) (*M* = mean age)	Brain measurements	Task at measurement
Cross-sectional studies						
Wang et al. (2011) [23]	KK SK	Expert meditators for ≥30 years; healthy		M: *n* = 10 (53.7 years)	1. CBF (fMRI)	t1: baseline 1 t2: control (counting while touching fingers with no sequence) t3: KK t4: SK t5: baseline 2
Lazar et al. (2000) [45]	KY	Expert meditators for ≥4 years; healthy		M:*n* = 5 (22–45)	1. Brain activation (brain fMRI)	t1: 6-min control (silently generating list of animals) t2: after 12 min of meditation
Khalsa et al. (2009) [46]	KK	Expert meditators; healthy		M: *n* = 11 (34.5; SD 13.5)	1. Brain SPECT	t1: rest, eyes closed t2: 12 min of meditation
Randomized controlled trials						
Eyre et al. (2016) [1•]	KK for 12 weeks	Novice meditators; MCI	MET	M: *n* = 14 (67.1; SD 9.5) C: *n* = 11 (67.8; SD 9.7)	1. Resting-state brain fMRI	t1: rest
Pomykala et al. (2012) [30]	KK for 8 weeks	Dementia caregivers; healthy	Music	M: *n* = 4 (56; SD 10.1) C: *n* = 5 (49.8; SD 3.9)	1. Brain PET	t1: rest, eyes open
Newberg et al. (2010b) [47]	KK for 8 weeks	Novice meditators; memory impairment	Music	M: *n* = 15 (64; SD 8) C: *n* = 5 (65; SD 10)	1. Brain SPECT	t1 and t3: preprogram and postprogram baseline (12 min of neutral informational CD) t2,M and t4,M: preprogram and postprogram meditation (12 min of meditation with meditation CD) t2,C and t4,C: preprogram and postprogram control (12 min of instrumental music CD)

Meditation types: *KK* Kirtan Kriya, *SK* Shabad Kriya, *KY* Kundalini Yoga; groups: *MCI* mild cognitive impairment, *MET* memory enhancement training; measurements: *CBF* cerebral blood flow, *fMRI* functional magnetic resonance imaging, *SPECT* single-photon emission computed tomography, *PET* positron emission tomography

Results showed that approximately 17 regions were consistently affected in both mindfulness- and active-based meditation studies. Common activations were shown in regions of the caudate, putamen, anterior cingulate cortex (ACC), thalamus, hippocampus, posterior cingulate cortex (PCC), precuneus, insula, fusiform gyrus, inferior frontal gyrus (IFG), several areas of the temporal gyrus, parietal areas, temporoparietal junction (TPJ), somatomotor cortex, orbitofrontal cortex, occipital area, and cerebellum (see Table 4 for results). Across these studies of meditative and yoga-based meditations, results showed less activation of the amygdala, an area that is well known for its role in processing emotionally salient stimuli [31••, 48].

**Table 4:** Brain areas associated with different meditative practices

Brain region	Reference
Brain areas common to both mindfulness- and active-based meditation practices	
Caudate	[23, 31••]
Putamen	[30, 40, 41, 45, 46]
Anterior cingulate	[1•, 18••, 31••, 40, 42, 45, 46]
Thalamus	[31••, 41, 43, 47]
Hippocampus	[23, 30, 31••, 40–43]
Posterior cingulate	[18••, 30, 31••, 42, 47]
Precuneus	[31••, 43, 44, 46, 47]
Insula	[18••, 23, 31••, 41, 44]
Fusiform gyrus	[31••, 46]
Inferior frontal gyrus	[30, 31••, 47]
Temporal area	[31••, 41, 45, 46]
Parietal area	[23, 43–46]
Temporoparietal junction	[31••, 42, 46]
Somatomotor cortex	[31••]
Orbitofrontal cortex	[31••, 41, 47]
Occipital lobe	[1•, 23, 31••, 43, 46]
Cerebellum	[30, 31••, 42–44]
Amygdala (deactivation)	[18••, 31••, 27]
Brain areas unique to mindfulness-based meditation practices	
Premotor area	[31••]
Mid-cingulate	[31••, 43]
Angular gyrus	[31••]
Somatosensory cortex (I and II)	[31••, 41, 42]
Brain areas unique to active-based practices	
Dorsolateral prefrontal cortex	[47]
Medial frontal cortex	[1•]
Superior temporal area	[23, 45, 46]
Paracentral lobule	[45, 46]
Precentral gyrus	[1•, 23, 45, 46]
Postcentral gyrus	[1•, 23, 45, 46]
Superior parietal lobule	[45–47]

### Brain Activations Unique to Mindfulness Meditative Practices

Four brain regions appeared to be unique to mindfulness-based meditations: the premotor area (PMA), the mid-cingulate, the angular gyrus (AG), and the primary and secondary somatosensory cortex (SSI and II).

### Brain Activations Unique to Active-Based Meditative Practices

Results from our review showed that approximately seven regions were uniquely affected by active-based meditations. These regions were the dorsolateral prefrontal cortex (DLPFC), the medial frontal cortex, the superior temporal area, the paracentral lobe, the precentral and postcentral gyrus, and the superior parietal lobule (SPL).

## Discussion

In this review, we focused on studies reporting the effects of different types of yogic meditations on the brain and distinguished between studies investigating mindfulness-based meditation (that ask participants to maintain an internal focus on the breath to achieve present-moment awareness) versus active-based styles (that involve chanting, active postures, visualization techniques, or partnered exercises).

Results from our review showed a pattern of neural regions that are consistently affected across meditation studies, namely areas involved in reward, motivation, and learning (the striatum); attention and memory (ACC, thalamus, and hippocampus); and sensory integration (insula), self-regulation (orbitofrontal cortex), and the DMN (PCC, precuneus, and TPJ). Common activations also appeared in the IFG, an area that is known for its role in empathy and language processing [49–51], and the fusiform gyrus, an area implicated in the recognition and process of faces [52] and more generally the recognition of objects that are salient or evocative in some way [53]. Also, the somatomotor cortex is involved in the sensory representation and the coordination of motor activities [54]. Collectively, these areas are targets of degeneration in healthy aging but also in other disorders. For example, a review of research studies by Mohan et al. (2016) showed that the DMN is disrupted in AD, PD, epilepsy, attention deficit hyperactivity disorder (ADHD), and mood disorders [33]. Another review of research found that connectivity and network integrity of the DMN decrease in healthy aging, but at a much more rapid pace in AD [55].

In combination with other research, results from the present review suggest that meditative practices commonly affect brain systems involved in attention, memory, conscious awareness, reward, and emotional regulation that are important and can be disrupted by cerebrovascular and neurodegenerative changes. Thus, as suggested by previous reviews, meditation may enhance cognitive and psychological health via neuroplastic effects on brain structures and circuits that influence attention, memory, and emotional regulation. Although these findings do not support stress buffering and relief models of meditation and yoga, it is likely that enhancement of these functions may indirectly alleviate stress. For example, by exerting greater self-control, individuals may better regulate their thoughts and emotions, thus possibly resulting in adaptive responses to stress.

### Brain Regions Unique to Mindfulness-Based Meditative Practices

A few neural regions emerged as unique to mindfulness practices: the mid-cingulate cortex (MCC), the angular gyrus (AG), the primary and secondary somatosensory cortex (SI and SII), and the PMA. The MCC is particularly interesting in the context of mindfulness meditation as it has been conceptualized as integrating emotionally salient interoceptive information that may ultimately result in improved movement–balance activity [56]. One study showed that the MCC was activated when subjects read emotionally evocative (versus neutral) stories and that its activation was correlated with self-reported feelings of “immersion” while reading [57]. Yet, other studies have shown the MCC’s involvement in environmental monitoring, response selection, and motor body orientation [58]. Due to the MCC’s role in integration of emotional information, movement, and behavior, some frameworks have suggested that it may play a key role in self-control [59–61]. This is consistent with studies suggesting that meditation exerts many of its benefits via enhanced self-regulation of emotion, thoughts, behaviors, and decision-making [62–64]. Thus, it is believed that, ultimately, this may result in more “conscious” or “conscientious” behavior for practitioners of mindfulness meditation [31••]. However, this may also confer the meditations’ benefits on mood disorders as well as ADD [33].

The AG has been implicated in awareness, understanding others’ intentions (theory of the mind), memory, and body awareness. Perhaps, most striking is that stimulation of the right AG induces out-of-body experiences [65]. As such, a recent review highlighted the AG’s role in integrating multisensory information to give sense to events, solve problems, adjust mental representations, and reorient attention to salient information [66]. The somatosensory cortex is one of the major hubs responsible for processing exteroceptive body information [67]. The human premotor cortex is involved in both motor and cognitive faculties. Its cognitive functions include space perception, action understanding and imitation, and learning [68], and its motor functions include the preparation for, coordination of, and sensory guidance of movement [69].

In sum, these results suggest that mindfulness meditations uniquely activated areas involved in sensory and emotional integration, self-control, body awareness, and movement. Thus, mindfulness practices may be especially useful in enhancing self-regulation and internal awareness, which may result in more conscious behaviors and lifestyles, and provide alleviation of mood disorders.

### Brain Regions Unique to Active-Based Meditative Practices

Our review of meditation studies also revealed several brain structures to be uniquely affected by active-based meditative practices, mainly regions involved in self-control, social cognition, language speech, tactile stimulation, sensorimotor integration, and motor function. These areas can broadly be thought of as subserving “willful acts” and movement, as well as social processes. For example, the dorsolateral prefrontal cortex (DLPFC) is well known for its role in cognitive and executive functions, and more specifically, it has been implicated in the evaluation of rewards, working memory, “sense of agency,” and self-control [70–74]. Dysregulation of DLPFC function has been widely shown in mood disorders, and thus, it appears to be a major target site for the treatment of depression [75–77]. Unique activation of the DLPFC resulting from active-based meditations is in line with numerous studies to date showing improvements in mood, depression, anxiety, and stress resulting from specific meditative practices that may involve chanting, active postures, and hand movements [78–80]. The medial pre frontal cortex (mPFC) was also shown to be uniquely related to active (versus mindful) meditation styles. It plays a major role in social cognition [81], most notably in making self-other judgments as shown in a meta-analysis of 107 studies [82].

Several key regions that subserve language, tactile stimulation, and movement were also shown to be uniquely activated for active-type meditative practices. These areas include the superior temporal lobe, an area of the brain that is involved in language, spatial processing, and social perceptions [83]; the paracentral lobule, involved in language and speech [84]; and the precentral gyrus, a brain region that is involved in articulation, speech, hand movements, and tactile stimulation [85, 86]. Also, the postcentral gyrus and SPL were shown to be unique to active-based meditations. The postcentral gyrus is largely involved in sensory and motor functions, primarily in the sense of touch [87]. This is consistent with the tasks asked of participants in active meditation styles, which involve tactile stimulation, specific hand postures, and moving of the fingers. The SPL is involved in attention and visual shifts, coordination of visual to motor information, and higher-order processes that give rise to the position of the body [88–92]. It is critical for sensorimotor integration by maintaining an internal representation of the body’s state [93] and plays a major role in the integration of auditory and visual signals [94]. As suggested by several studies implicating the SPL, spatial, working, and episodic memories are necessary for maintaining internal body representations [95, 96]; thus, it is closely related to working memory as well.

### The Use of Meditation for Promoting Healthy Aging

Meditative practices may be especially useful in aging adults for several reasons. First, these low-impact practices are less strenuous compared to many other exercises that can be used in older adults with physical limitations without significant risk of adverse events. Many of the practices can be adapted to a sitting or lying down position. Second, meditation appears to improve mood, coping with chronic stress, and provides relief of chronic pain [97]. Finally, meditation offers safe, non-pharmacologic alternative or adjunctive treatments, thus reducing polypharmacy.

The emerging evidence points to the “brain fitness” effects of meditative practices that can support cognitive abilities, memory, somatosensory, and motor functions [98–100]. This may be particularly beneficial in aging adults at-risk for cognitive decline. It is also interesting to note that many of the circuits affected by meditation are also implicated in social processes including empathy [101, 102]. Recruitment of these processes may enhance well-being indirectly by facilitating high-quality social interactions [103, 104]. They may also enhance caregiving and related behaviors, which play a critical role in attachment relationships where one individual is highly dependent on the other, such as parent–infant relationships and those where one spouse may be ill and in need of care [105, 106]. More broadly, social processes related to cooperation, empathy, and altruism are thought to be involved in the evolution and thriving of the species [107–111].

By parsing out the neural networks affected by these different meditation techniques, we propose to better understand how meditation works and, more importantly, to determine which meditation styles may be useful for specific functions. For example, a recent pilot study that compared resting-state brain activity among a group of individuals reporting mild cognitive issues (MCI) that were randomly assigned to a daily brief yogic meditation (Kirtan Kirya) with once-weekly KY class condition versus a rigorous “memory enhancement training” control condition showed significant correlations between improvements in clinical measures of verbal memory and visual–spatial memory performance in the yoga group with connectivity in the DMN and the language processing network, respectively [1•]. Thus, this study showed that an active meditation practice may be as effective as a “gold standard” memory enhancement training program for improving memory and brain connectivity, which may be particularly useful for older adults with mild cognitive impairment (MCI) and those experiencing acute or chronic stress deleterious for cognitive and physical functioning.

## Conclusions

Meditation has been used to improve awareness and well-being for thousands of years, but given recent technological advances and the growing popularity of these practices in the West, research studies are increasingly examining their efficacy and underlying mechanisms. Our review suggests that meditation influences brain systems involved in attention, awareness, memory, sensory integration, and the cognitive regulation of emotion. More nuanced results were also found showing unique recruitment for mindfulness practices, in brain regions that regulate body awareness and higher-order cognitive functions. Active-based meditative approaches showed unique effects too, in areas that mainly affect social processes (such as speech, language, empathy, and facial processing) and self-regulation. Collectively, these results suggest that meditation may be especially useful for brain fitness in aging adults as they provide enhancements of higher cognitive functions and social cognition, attention, memory, movement, and emotional regulation that can help in preventing mood, physical, and cognitive disorders of aging.

## References

[1] Eyre HA, Acevedo B, Yang H (2016). Changes in neural connectivity and memory following a yoga intervention for older adults: a pilot study. J Alzheimers Dis.

[2] Jain FA, Nazarian N, Lavretsky H (2014). Feasibility of central meditation and imagery therapy for dementia caregivers. Int J Geriatr Psychiatry..

[3] Siddarth D, Siddarth P, Lavretsky H (2014). An observational study of the health benefits of yoga or tai chi compared with aerobic exercise in community-dwelling middle-aged and older adults. Am J Geriatr Psychiatry.

[4] Abbott R, Lavretsky H (2013). Tai Chi and Qigong for the treatment and prevention of mental disorders. Psychiatr Clin North Am..

[5] Black DS, Cole SW, Irwin MR (2013). Yogic meditation reverses NF-κB and IRF-related transcriptome dynamics in leukocytes of family dementia caregivers in a randomized controlled trial. Psychoneuroendocrinology.

[6] Barnes PM, Powell-Griner E, McFann K, Nahin RL (2004). Complementary and alternative medicine use among adults: United States, 2002. Adv Data.

[7] United Nations, Department of Economic and Social Affairs, Population Division. (2015). World population ageing 2015 (ST/ESA/SER.A/390).

[8] World Health Organization (2010). Recommended population levels of physical activity for health. Global recommendations on physical activity for health.

[9] Violan C, Foguet-Boreu Q, Flores-Mateo G, Salisbury C, Blom J, Freitag M, Glynn L, Muth C, Valderas JM (2014). Prevalence, determinants and patterns of multimorbidity in primary care: a systematic review of observational studies. PLoS One.

[10] Shah BM, Hajjar ER (2012). Polypharmacy, adverse drug reactions, and geriatric syndromes. Clin Geriatr Med.

[11] Clarke TC, Black LI, Stussman BJ, Barnes PM, Nahin RL (2015). Trends in the use of complementary health approaches among adults: United States, 2002–2012. Natl Health Stat Report.

[12] Eyre H, Baune B, Lavretsky H (2015). Clinical advances in geriatric psychiatry: a focus on prevention of mood and cognitive disorders. Psychiatr Clin North Am.

[13] Lavretsky H (2009). Complementary and alternative medicine use for treatment and prevention of late-life mood and cognitive disorders. Aging Health.

[14] McCaffrey AM, Pugh GF, O’Connor BB (2007). Understanding patient preference for integrative medical care: results from patient focus groups. J Gen Intern Med.

[15] Meeks TW, Wetherell JL, Irwin MR, Redwine LS, Jeste DV (2007). Complementary and alternative treatments for late-life depression, anxiety, and sleep disturbance: a review of randomized controlled trials. J Clin Psychiatry.

[16] Russinova Z, Cash D, Wewiorski NJ (2009). Toward understanding the usefulness of complementary and alternative medicine for individuals with serious mental illnesses: classification of perceived benefits. J Nerv Ment Dis.

[17] Su D, Li L (2011). Trends in the use of complementary and alternative medicine in the United States: 2002-2007. J Health Care Poor Underserved.

[18] Tang YY, Hölzel BK, Posner MI (2015). The neuroscience of mindfulness meditation. Nat Rev Neurosci.

[19] Brewer JA, Worhunsky PD, Gray JR (2011). Meditation experience is associated with differences in default mode network activity and connectivity. Proc Natl Acad Sci U S A.

[20] Leung MK, Chan CC, Yin J (2013). Increased grey matter volume in the right angular and posterior parahippocampal gyri in loving- kindness meditators. Soc Cogn Affect Neurosci.

[21] Davidson RJ, Goleman DJ (1977). The role of attention in meditation and hypnosis: a psychobiological perspective on transformations of consciousness. Int J Clin Exp Hypn.

[22] Newberg AB, Iversen J (2003). The neural basis of the complex mental task of meditation: neurotransmitter and neurochemical considerations. Med Hypotheses.

[23] Wang DJ, Rao H, Korczykowski M (2011). Cerebral blood flow changes associated with different meditation practices and perceived depth of meditation. Psychiatry Res.

[24] Ross A, Thomas S (2010). The health benefits of yoga and exercise: a review of comparison studies. J Altern Complement Med.

[25] Vedamurthachar A, Janakiramaiah N, Hegde JM (2006). Antidepressant efficacy and hormonal effects of Sudarshana Kriya yoga (SKY) in alcohol dependent individuals. J Affect Disord.

[26] Chandwani KD, Perkins G, Nagendra HR (2014). Randomized, controlled trial of yoga in women with breast cancer undergoing radiotherapy. J Clin Oncol.

[27] Corey SM, Epel E, Schembri M (2014). Effect of restorative yoga vs. stretching on diurnal cortisol dynamics and psychosocial outcomes in individuals with the metabolic syndrome: the PRYSMS randomized controlled trial. Psychoneuroendocrinology.

[28] Sieverdes JC, Mueller M, Gregoski MJ (2014). Effects of Hatha yoga on blood pressure, salivary α-amylase, and cortisol function among normotensive and prehypertensive youth. J Altern Complement Med.

[29] Yoshihara K, Hiramoto T, Oka T (2014). Effect of 12 weeks of yoga training on the somatization, psychological symptoms, and stress-related biomarkers of healthy women. Biopsychosoc Med.

[30] Pomykala KL, Silverman DH, Geist CL (2012). A pilot study of the effects of meditation on regional brain metabolism in distressed dementia caregivers. Aging Health.

[31] Fox KC, Nijeboer S, Dixon ML (2014). Is meditation associated with altered brain structure? A systematic review and meta-analysis of morphometric neuroimaging in meditation practitioners. Neurosci Biobehav Rev.

[32] Li HJ, Hou XH, Liu HH, Yue CL, He Y, Zuo XN (2015). Toward systems neuroscience in mild cognitive impairment and Alzheimer’s disease: a meta-analysis of 75 fMRI studies. Hum Brain Mapp.

[33] Mohan A, Roberto AJ, Mohan A, Lorenzo A, Jones K, Carney MJ, Liogier-Weyback L, Hwang S, Lapidus KA (2016). The significance of the default mode network (DMN) in neurological and neuropsychiatric disorders: a review. Yale J Biol Med.

[34] Hölzel BK, Carmody J, Vangel M (2011). Mindfulness practice leads to increases in regional brain gray matter density. Psychiatry Res.

[35] Streeter CC, Gerbarg PL, Saper RB (2012). Effects of yoga on the autonomic nervous system, gamma-aminobutyric-acid, and allostasis in epilepsy, depression, and post-traumatic stress disorder. Med Hypotheses.

[36] Streeter CC, Whitfield TH, Owen L (2010). Effects of yoga versus walking on mood, anxiety, and brain GABA levels: a randomized controlled MRS study. J Altern Complement Med.

[37] Kjaer TW, Bertelsen C, Piccini P (2002). Increased dopamine tone during meditation-induced change of consciousness. Cogn Brain Res.

[38] Schultz W (2002). Getting formal with dopamine and reward. Neuron.

[39] Berridge KC, Robinson TE (1998). What is the role of dopamine in reward: hedonic impact, reward learning, or incentive salience?. Brain Res Rev.

[40] Chiesa A, Serretti A (2010). A systematic review of neurobiological and clinical features of mindfulness meditations. Psychol Med.

[41] Ott U, Hölzel BK, Vaitl D, Walach H, Schmidt S, Jonas WB (2011). Brain structure and meditation: how spiritual practice shapes the brain. Neuroscience, consciousness and spirituality.

[42] Marciniak R, Sheardova K, Čermáková P (2014). Effect of meditation on cognitive functions in context of aging and neurodegenerative diseases. Front Behav Neurosci.

[43] Luders E, Cherbuin N, Kurth F (2015). Forever young (er): potential age-defying effects of long-term meditation on gray matter atrophy. Front Psychol.

[44] Newberg AB, Wintering N, Waldman MR (2010). Cerebral blood flow differences between long-term meditators and non-meditators. Conscious Cogn.

[45] Lazar SW, Bush G, Gollub RL (2000). Functional brain mapping of the relaxation response and meditation. Neuroreport.

[46] Khalsa DS, Amen D, Hanks C (2009). Cerebral blood flow changes during chanting meditation. Nucl Med Commun.

[47] Newberg AB, Wintering N, Khalsa DS (2010). Meditation effects on cognitive function and cerebral blood flow in subjects with memory loss: a preliminary study. J Alzheimers Dis.

[48] Phan KL, Wager T, Taylor SF (2002). Functional neuroanatomy of emotion: a meta-analysis of emotion activation studies in PET and fMRI. NeuroImage.

[49] Costafreda SG, Fu CH, Lee L (2006). A systematic review and quantitative appraisal of fMRI studies of verbal fluency: role of the left inferior frontal gyrus. Hum Brain Mapp.

[50] Mazza M, Tempesta D, Pino MC (2015). Neural activity related to cognitive and emotional empathy in post-traumatic stress disorder. Behav Brain Res.

[51] Fujino J, Takahashi H, Miyata J (2014). Impaired empathic abilities and reduced white matter integrity in schizophrenia. Prog Neuro-Psychopharmacol Biol Psychiatry.

[52] Bokde ALW, Lopez-Bayo P, Meindl T (2006). Functional connectivity of the fusiform gyrus during a face-matching task in subjects with mild cognitive impairment. Brain.

[53] Garoff RJ, Slotnick SD, Schacter DL (2005). The neural origins of specific and general memory: the role of the fusiform cortex. Neuropsychologia.

[54] Dresler M, Koch SP, Wehrle R (2011). Dreamed movement elicits activation in the sensorimotor cortex. Curr Biol.

[55] Dennis EL, Thompson PM (2014). Functional brain connectivity using fMRI in aging and Alzheimer’s disease. Neuropsychol Rev.

[56] Hoffstaedter F, Grefkes C, Caspers S (2014). The role of anterior midcingulate cortex in cognitive motor control. Hum Brain Mapp.

[57] Hsu CT, Conrad M, Jacobs AM (2014). Fiction feelings in Harry Potter: haemodynamic response in the mid-cingulate cortex correlates with immersive reading experience. Neuroreport.

[58] Taylor KS, Seminowicz DA, Davis KD (2009). Two systems of resting state connectivity between the insula and cingulate cortex. Hum Brain Mapp.

[59] Bush G, Luu P, Posner MI (2000). Cognitive and emotional influences in anterior cingulate cortex. Trends Cogn Sci.

[60] Posner MI, Rothbart MK, Sheese BE (2007). The anterior cingulate gyrus and the mechanism of self-regulation. Cogn Affect Behav Neurosci.

[61] Allman JM, Hakeem A, Erwin JM (2001). The anterior cingulate cortex. Ann N Y Acad Sci.

[62] Condon P, Desbordes G, Miller WB (2013). Meditation increases compassionate responses to suffering. Psychol Sci.

[63] Grant JA, Courtemanche J, Duerden EG (2010). Cortical thickness and pain sensitivity in Zen meditators. Emotion.

[64] Shackman AJ, Salomons TV, Slagter HA (2011). The integration of negative affect, pain and cognitive control in the cingulate cortex. Nat Rev Neurosci..

[65] Blanke O, Ortigue S, Landis T (2002). Neuropsychology: stimulating illusory own-body perceptions. Nature.

[66] Seghier ML (2013). The angular gyrus multiple functions and multiple subdivisions. Neuroscientist.

[67] Farb NA, Segal ZV, Anderson AK (2013). Mindfulness meditation training alters cortical representations of interoceptive attention. Soc Cogn Affect Neurosci.

[68] Kantak SS, Stinear JW, Buch ER (2012). Rewiring the brain potential role of the premotor cortex in motor control, learning, and recovery of function following brain injury. Neurorehabil Neural Repair.

[69] Wise SP (1985). The primate premotor cortex: past, present, and preparatory. Annu Rev Neurosci.

[70] Balconi M (2013). Dorsolateral prefrontal cortex, working memory and episodic memory processes: insight through transcranial magnetic stimulation techniques. Neurosci Bull.

[71] Barbey AK, Koenigs M, Grafman J (2013). Dorsolateral prefrontal contributions to human working memory. Cortex.

[72] Brunoni AR, Boggio PS, De Raedt R (2014). Cognitive control therapy and transcranial direct current stimulation for depression: a randomized, double-blinded, controlled trial. J Affect Disord.

[73] Khalighinejad N, Di Costa S, Haggard P (2016). Endogenous action selection processes in dorsolateral prefrontal cortex contribute to sense of agency: a meta-analysis of tDCS studies of ‘intentional binding’. Brain Stimul.

[74] Sperduti M, Delaveau P, Fossati P (2011). Different brain structures related to self-and external-agency attribution: a brief review and meta-analysis. Brain Struct Funct.

[75] Bosch OG, Rihm JS, Scheidegger M (2013). Sleep deprivation increases dorsal nexus connectivity to the dorsolateral prefrontal cortex in humans. Proc Natl Acad Sci U S A.

[76] Koenigs M, Grafman J (2009). The functional neuroanatomy of depression: distinct roles for ventromedial and dorsolateral prefrontal cortex. Behav Brain Res.

[77] Schutter DJ (2009). Antidepressant efficacy of high-frequency transcranial magnetic stimulation over the left dorsolateral prefrontal cortex in double-blind sham-controlled designs: a meta-analysis. Psychol Med.

[78] Innes KE, Selfe TK, Brown CJ (2012). The effects of meditation on perceived stress and related indices of psychological status and sympathetic activation in persons with Alzheimer’s disease and their caregivers: a pilot study. Evid Based Complement Alternat Med.

[79] Lavretsky H, Epel ES, Siddarth P (2013). A pilot study of yogic meditation for family dementia caregivers with depressive symptoms: effects on mental health, cognition, and telomerase activity. Int J Geriatr Psychiatry.

[80] Moss AS, Wintering N, Roggenkamp H (2012). Effects of an 8-week meditation program on mood and anxiety in patients with memory loss. J Altern Complement Med.

[81] Amodio DM, Frith CD (2006). Meeting of minds: the medial frontal cortex and social cognition. Nat Rev Neurosci.

[82] Denny BT, Kober H, Wager TD (2012). A meta-analysis of functional neuroimaging studies of self-and other judgments reveals a spatial gradient for mentalizing in medial prefrontal cortex. J Cogn Neurosci.

[83] Hein G, Knight RT (2008). Superior temporal sulcus—it’s my area: or is it?. J Cogn Neurosci.

[84] Imamura T, Tsuburaya K (1992). Absence of neurobehavioral disturbance in a focal lesion of the left paracentral lobule. Behav Neurol.

[85] Baldo JV, Wilkins DP, Ogar J (2011). Role of the precentral gyrus of the insula in complex articulation. Cortex.

[86] Luders E, Kurth F, Mayer EA (2012). The unique brain anatomy of meditation practitioners: alterations in cortical gyrification. Front Hum Neurosci.

[87] Iwamura Y, Tanaka M (1996). Representation of reaching and grasping in the monkey postcentral gyrus. Neurosci Lett.

[88] Caminiti R, Ferraina S, Johnson PB (1996). The sources of visual information to the primate frontal lobe: a novel role for the superior parietal lobule. Cereb Cortex.

[89] Corbetta M, Shulman GL, Miezin FM (1995). Superior parietal cortex activation during spatial attention shifts and visual feature conjunction. Science.

[90] Galletti C, Fattori P, Kutz DF (1997). Arm movement-related neurons in the visual area V6A of the macaque superior parietal lobule. Eur J Neurosci.

[91] Sakata H, Takaoka Y, Kawarasaki A (1973). Somatosensory properties of neurons in the superior parietal cortex (area 5) of the rhesus monkey. Brain Res.

[92] Vandenberghe R, Gitelman DR, Parrish TB (2001). Functional specificity of superior parietal mediation of spatial shifting. NeuroImage.

[93] Wolpert DM, Goodbody SJ, Husain M (1998). Maintaining internal representations: the role of the human superior parietal lobe. Nat Neurosci.

[94] Molholm S, Sehatpour P, Mehta AD (2006). Audio-visual multisensory integration in superior parietal lobule revealed by human intracranial recordings. J Neurophysiol.

[95] Coull JT, Frith CD (1998). Differential activation of right superior parietal cortex and intraparietal sulcus by spatial and nonspatial attention. NeuroImage.

[96] Koenigs M, Barbey AK, Postle BR (2009). Superior parietal cortex is critical for the manipulation of information in working memory. J Neurosci.

[97] Field T (2011). Yoga clinical research review. Complement Ther Clin Pract.

[98] Gard T, Taquet M, Dixit R (2015). Greater widespread functional connectivity of the caudate in older adults who practice kripalu yoga and vipassana meditation than in controls. Front Hum Neurosci.

[99] Gard T, Taquet M, Dixit R (2014). Fluid intelligence and brain functional organization in aging yoga and meditation practitioners. Front Aging Neurosci.

[100] Gard T, Hölzel BK, Lazar SW (2014). The potential effects of meditation on age-related cognitive decline: a systematic review. Ann N Y Acad Sci.

[101] Acevedo BP, Aron A, Fisher HE (2011). Neural correlates of long-term intense romantic love. Soc Cogn Affect Neurosci.

[102] Acevedo BP, Aron EN, Aron A (2014). The highly sensitive brain: an fMRI study of sensory processing sensitivity and response to others’ emotions. Brain Behav.

[103] Kiecolt-Glaser JK, Page GG, Marucha PT (1998). Psychological influences on surgical recovery: perspectives from psychoneuroimmunology. Am Psychol.

[104] Riehl-Emde A, Thomas V, Willi J (2003). Love: an important dimension in marital research and therapy. Fam Process.

[105] Bowlby J (1969). Attachment and loss: vol. 1. Attachment.

[106] Acevedo BP, Poulin M, Brown LL. Human pair-bonding: neural and genetic correlates reveal phylogenetically conserved brain systems mediating selfless love in marriage.

[107] Batson CD (1983). Sociobiology and the role of religion in promoting prosocial behavior: an alternative view. J Pers Soc Psychol.

[108] Eisenberg N, Miller PA (1987). The relation of empathy to prosocial and related behaviors. Psychol Bull.

[109] Silk JB, House BR (2011). Evolutionary foundations of human prosocial sentiments. Proc Natl Acad Sci U S A.

[110] Tomasello M, Melis AP, Tennie C (2012). Two key steps in the evolution of human cooperation. Curr Anthropol.

[111] Trivers RL (1971). The evolution of reciprocal altruism. Q Rev Biol.

